# Impact of anesthesia on pathophysiology and mortality following subarachnoid hemorrhage in rats

**DOI:** 10.1186/2040-7378-4-5

**Published:** 2012-03-13

**Authors:** Konstantin Hockel, Raimund Trabold, Karsten Schöller, Elisabeth Török, Nikolaus Plesnila

**Affiliations:** 1 Laboratory of Experimental Neurosurgery, Institute for Surgical Research, Munich, Germany; 2 Department of Neurosurgery, Munich, Germany; 3 Institute for Stroke and Dementia Research, University of Munich Medical Center - Grosshadern, Ludwig-Maximilians-University, Munich, Germany

**Keywords:** Subarachnoid hemorrhage, Rat, Experimental, Anesthesia, Isoflurane, Chloral hydrate, Medetomidine, Brain edema, Cerebral blood flow, Re-bleeding

## Abstract

**Background:**

Anesthesia is indispensable for in vivo research but has the intrinsic potential to alter study results. The aim of the current study was to investigate the impact of three common anesthesia protocols on physiological parameters and outcome following the most common experimental model for subarachnoid hemorrhage (SAH), endovascular perforation.

**Methods:**

Sprague-Dawley rats (n = 38) were randomly assigned to (1) chloral hydrate, (2) isoflurane or (3) midazolam/medetomidine/fentanyl (MMF) anesthesia. Arterial blood gases, intracranial pressure (ICP), mean arterial blood pressure (MAP), cerebral perfusion pressure (CPP), and regional cerebral blood flow (rCBF) were monitored before and for 3 hours after SAH. Brain water content, mortality and rate of secondary bleeding were also evaluated.

**Results:**

Under baseline conditions isoflurane anesthesia resulted in deterioration of respiratory parameters (arterial pCO_2 _and pO_2_) and increased brain water content. After SAH, isoflurane and chloral hydrate were associated with reduced MAP, incomplete recovery of post-hemorrhagic rCBF (23 ± 13% and 87 ± 18% of baseline, respectively) and a high anesthesia-related mortality (17 and 50%, respectively). Anesthesia with MMF provided stable hemodynamics (MAP between 100-110 mmHg), high post-hemorrhagic rCBF values, and a high rate of re-bleedings (> 50%), a phenomenon often observed after SAH in humans.

**Conclusion:**

Based on these findings we recommend anesthesia with MMF for the endovascular perforation model of SAH.

## Background

Experimental animal models are widely used to study the pathophysiology of subarachnoid hemorrhage (SAH) [1-4]. The induction of experimental SAH is performed by invasive surgical techniques which require continuous multimodal monitoring and adequate sedation and analgesia. Ventilation and, hence, blood gases can be controlled by intubation and mechanical ventilation [5], however, other parameters which are known to critically determine the outcome following experimental SAH such as intracranial pressure (ICP), mean arterial blood pressure (MAP) and regional cerebral blood flow (rCBF) [1,2,6-9] may well be influenced by the applied anesthetic protocol as already demonstrated for experimental models of cerebral ischemia [5] and traumatic brain injury [10,11].

Various inhaled or injectable anesthetics have been used in experimental SAH, e.g. halothane, isoflurane [alone or in combination with nitrous oxide (N_2_O)] [1,3,4,12,13], chloral hydrate, barbiturates [14-16], ketamine/xylazine or midazolam/medetomedine/fentanyl (MMF) [17-19]. Although experimental SAH models are known to suffer from some variability [2,3,9], little is know to which degree the used anesthetic protocol contributes to this phenomenon. Therefore, we investigated the effect of three well established and frequently used standard anesthesia protocols, namely chloral hydrate, isoflurane, and MMF on animal physiology, mortality and brain water content before and after experimental SAH in rats. The ultimate aim of the study was to determine how anesthesia influences the outcome of experimental SAH and, if significant differences are found between different anesthesia protocols, which protocol has the least influence on the pathophysiology induced by the endovascular perforation model, the most frequently used and the presumably most clinically relevant model of subarachnoid hemorrhage.

## Methods

In this study we used 38 male Sprague-Dawley rats (250-300 g body weight), purchased from Charles River Laboratories, Sulzfeld, Germany. Eight animals died during experiments (see below), i.e. 30 animals were included in the final analysis. Animals had free access to pellet food until 12 hours prior to surgery. Water was accessible at all time. All experiments were approved by the Ethics Committee of the District Government of Upper Bavaria, Germany.

### Anesthesia

Anesthesia protocols were chosen based on the frequency of their use with the endovascular perforation model of SAH (e.g. isoflurane), on their availability (e.g. halothane was not included in the analysis since its production for human use was discontinued resulting in a reduced availability for many laboratories), on their known small influence of cerebral blood flow (chloral hydrate and midazolam/medetomidine/fentanyl), on the ease of their termination (isoflurane and midazolam/medetomidine/fentanyl), and on their level of standardization in experimental and veterinary medicine (all three protocols).

Dose regiments were based on the scientific and veterinary literature and our own experience during the past 5 (midazolam/medetomidine/fentanyl) to 20 (chloral hydrate and isoflurane) years. A similar level of sedation between the three protocols was achieved by using the lowest dose necessary to reach surgical anesthesia (as verified by the tail pinch test and concomitant observation of withdrawal reflexes and systemic blood pressure).

Anesthesia was induced by placing animals in a chamber with 4% isoflurane for 2-3 minutes. Thereafter rats were randomized to one of the three standard anesthesia protocols: (1) Chloral hydrate (1 ml/100 g body weight of a 3.6% solution) was injected intraperitoneally (i.p.). Animals were intubated and ventilated with 70% air and 30% O_2 _under control of ventilation pressure and rate (Small animal ventilator KTR-4, Hugo Sachs Elektronik, Germany). A third of the initial dosage was applied every 60 minutes in order to maintain anesthesia. (2) Animals were ventilated with 2% isoflurane in 70% N_2_O and 30% O_2_. (3) Midazolam (2 mg/kg), medetomidine (0.15 mg/kg) and fentanyl (0.0075 mg/kg) were given by intraperitoneal injection. Animals were ventilated with 70% air and 30% O_2_. A third of the initial dosage was applied for maintenance of anesthesia every 40 minutes. Atropine (150 mg/kg bodyweight) was injected subcutaneously in all experimental groups to inhibit salivary secretion and induce bronchodilation.

### Monitoring

The tail artery was canulated for continuous measurement of mean arterial blood pressure (MAP) and for blood sampling throughout the experiment. Arterial blood gases, pH, serum glucose and lactate were analyzed every 30 minutes. Ventilation rate (in ventilations per minute = vpm) and pressure were adapted in order to maintain physiological arterial pCO_2 _values (35-45 mmHg). Temporal muscle and rectal probes were used to monitor brain and body temperature, respectively. A thermostatically regulated, feedback-controlled heating pad and lamp were used to maintain rectal and brain temperature at 37°C.

Intracranial pressure (ICP) was continuously measured using a Codman ICP microsensor (Johnson & Johnson Medical Limited, Berkshire, UK). After drilling a burr hole over the right parietal cortex under constant cooling, the probe was advanced 2 mm into the brain and fixed with dental cement. Cerebral perfusion pressure (CPP) was calculated using the following formula: CPP = MAP - ICP.

A 2-channel laser-Doppler flowmeter (LDF; MBF3D, Moor Instruments Ltd.) was used for continuous monitoring of regional cerebral blood flow (rCBF) in the territory of the middle cerebral artery (MCA) of both hemispheres as previously described [4,13].

### Induction of SAH

SAH was induced by endovascular puncture, one of the most widely used models for experimental SAH [1-3], as previously described [4]. Briefly, a 3-0 monofilament was advanced via the external carotid artery (ECA) into the internal carotid artery (ICA) until increase of ICP and bilateral decrease of rCBF indicated SAH. Subsequently, the suture was withdrawn and the ECA ligated.

### Experimental groups

In a first series of experiments blood gases, MAP, ICP, CPP and rCBF were continuously monitored for 3 hours under physiological conditions in all three experimental groups (n = 5 each). Afterwards animals were sacrificed for quantification of brain water content. In a second series animals were randomly assigned to one of the three anesthesia groups (n = 5 each) and SAH was induced. All physiologic parameters were recorded under baseline conditions and continuously over 3 hours after SAH. At the end of observation time anesthesia was terminated and animals were tested for signs of awakening, i.e. adequate spontaneous respiration. Animals that died during surgery or exhibited excessive re-bleedings were replaced until the final group size was n = 5 in all groups. Subsequently, animals were re-anesthetized and sacrificed for quantification of brain water content.

### Quantification of brain water content

Brains were removed and the hemispheres were separated and weighed to assess their wet weight (WW). Thereafter, the hemispheres were dried for 24 h at 110°C and their dry weight (DW) was determined. Hemispheric water content (%) was calculated using the following formula: ((WW-DW)/WW) × 100.

### Statistical analysis

Statistical analysis was performed with SigmaStat 3.1 (SPSS Science Inc., Chicago, IL, USA). Blood gas parameters were analyzed with Kruskal-Wallis - ANOVA on ranks followed by Student-Newman-Keuls as post hoc test, and for physiologic parameters ANOVA on ranks/Dunn's Method was applied. Two points in time of the same group were compared with Friedman - repeated measures ANOVA on ranks. Statistical significance of results was assumed at p < 0.05 and a power of > 0.8. Data are presented as means ± SEM if not otherwise indicated.

## Results

All three anesthesia protocols provided sufficient sedation and analgesia for surgical intervention - the animals showed no signs of untimely awakening or discomfort at any time. Pinching of the hind paw did not trigger a withdrawal reflex or an increase in blood pressure.

### Ventilation rate and blood gases

Under physiological conditions anesthesia with isoflurane required a significant increase of ventilation rate during the 180 minutes of observation to keep PaCO_2 _within normal limits (58 ± 2 vpm vs. 27 ± 1 vpm in rat anesthetized with MMF; *p *< 0.05; Table 1).

**Table 1:** Physiological parameters during surgical preparation, before, and after SAH

			physiological conditions			after SAH		
		Preparation	0 min	60 min	180 min	0 min	60 min	180 min
Ventilation rate (v/min)	chloral hydrate	36 ± 1	36 ± 2	46 ± 3*	47 ± 3*	37 ± 1	39 ± 2	42 ± 4*
	isoflurane	35 ± 2	44 ± 4*	51 ± 2*	58 ± 2*	44 ± 2	43 ± 2	47 ± 3*
	MMF	32 ± 2	30 ± 2	29 ± 2	27 ± 1	35 ± 3	35 ± 4	34 ± 3
Art. pCO_2 _(mmHg)	chloral hydrate	36.9 ± 2.9	41.6 ± 2.8	41.8 ± 2.5	40.8 ± 3.4	40.5 ± 1.7	41.7 ± 2.2	43.2 ± 3.0
	isoflurane	44.2 ± 1.9	45.1 ± 3.1	41.7 ± 4.0	42.9 ± 2.7	38.6 ± 0.8	44.1 ± 2.0	42.4 ± 2.9
	MMF	40.3 ± 3.1	37.1 ± 1.2	34.5 ± 2.4	37.8 ± 3.3	41.7 ± 0.6	44.3 ± 2.3	43.3 ± 3.1
Art. pO_2 _(mmHg)	chloral hydrate	136.6 ± 4.1	120.3 ± 3.4	116.0 ± 6.3#	113.1 ± 4.7#	125.1 ± 6.8#	116.5 ± 7.1#	109.6 ± 9.4
	isoflurane	121.1 ± 4.2	105.1 ± 7.8	89.6 ± 2.8	86.4 ± 3.9	99.8 ± 5.3	92.3 ± 3.1	90.1 ± 5.6
	MMF	125.6 ± 9.5	122.2 ± 9.7	135.6 ± 8.8#	135.4 ± 6.7#	105.2 ± 8.0	106.2 ± 6.9#	116.1 ± 9.5
pH	chloral hydrate	7.29 ± 0.03	7.30 ± 0.02	7.29 ± 0.02	7.28 ± 0.03	7,34 ± 0,02	7.33 ± 0.01	7,28 ± 0,02
	isoflurane	7.29 ± 0.02	7.32 ± 0.05	7.33 ± 0.04	7.35 ± 0.03	7,33 ± 0,01	7.29 ± 0.01	7,31 ± 0,03
	MMF	7.34 ± 0.07	7.37 ± 0.01	7.35 ± 0.02	7.35 ± 0.01	7,37 ± 0,02	7.34 ± 0.04	7,35 ± 0,01
Glucose (mg/dl)	chloral hydrate	196 ± 24	231 ± 20	169 ± 14	115 ± 16	186 ± 27	162 ± 23	138 ± 18
	isoflurane	165 ± 20	158 ± 20	120 ± 10	114 ± 6	131 ± 15	121 ± 9	128 ± 8
	MMF	196 ± 51	191 ± 37	156 ± 23	174 ± 26	208 ± 43	173 ± 17	181 ± 24
Lactate **(**mmol/l)	chloral hydrate	1.0 ± 0.1	1.1 ± 0.1	0.8 ± 0.1	0.6 ± 0.1	1.2 ± 0.3	1.4 ± 0.3	0.7 ± 0.1
	isoflurane	1.0 ± 0.1	1.3 ± 0.1	1.4 ± 0.2	1.5 ± 0.3	1.2 ± 0.3	0.8 ± 0.1	1.0 ± 0.0
	MMF	0.5 ± 0.1	0.6 ± 0.0	0.7 ± 0.1	0.8 ± 0.2	0.6 ± 0.0	1.1 ± 0.1	0.8 ± 0.2

After SAH induction, anesthesia with isoflurane and chloral hydrate required an increase of the ventilation rate that reached statistical significance (*p *< 0.05) compared to MMF after 180 minutes (47 ± 3, 42 ± 4, and 34 ± 3 vpm, respectively) (Table 1). Ventilation pressure was not different between groups (12.6 ± 0.2, 12.7 ± 0.2 and 12.6 ± 0.4 cmH_2_O for chloral hydrate, isoflurane and MMF, respectively).

Arterial pCO_2 _and pH were not significantly different between the three anesthesia protocols under physiological conditions or after SAH induction. Arterial pO_2 _in the isoflurane group, however, decreased significantly during the course of the experiment (*p *< 0.05) and was significantly lower compared to chloral hydrate and MMF (*p *< 0.05) after 60 and 180 minutes under physiological conditions (116.0 ± 6.3, 89.6 ± 2.8, and 135.6 ± 8.8 mmHg for chloral hydrate, isoflurane and MMF, respectively). Accordingly, in SAH groups a decline in arterial pO_2 _was observed for isoflurane (*p *< 0.05), with values significantly different from the chloral hydrate and MMF group at 60 minutes after SAH (Table 1).

### ICP, MAP, CPP, and rCBF

**Figure 1:**
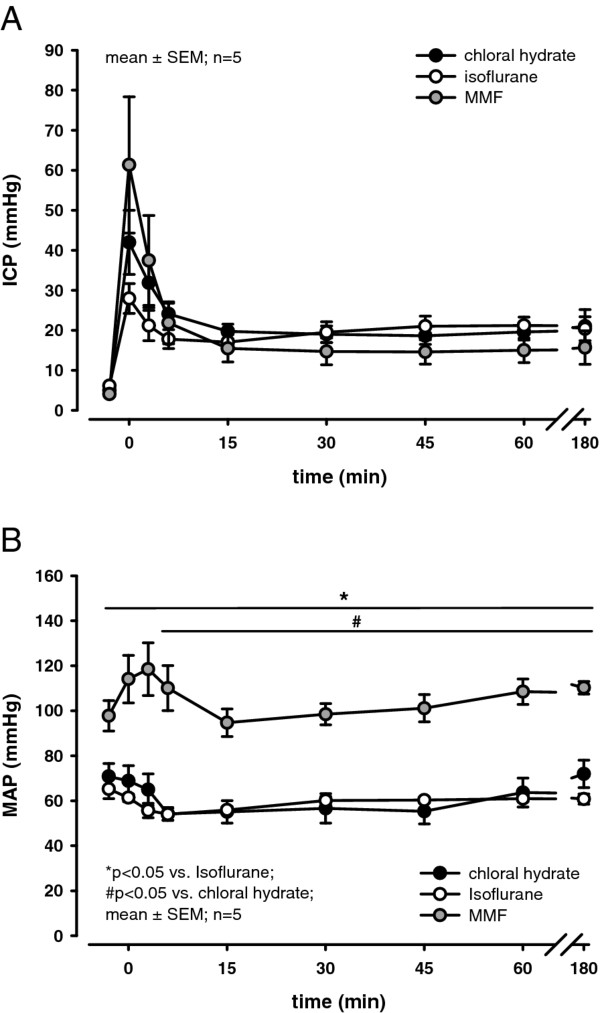
**(A) Time course of ICP until 180 minutes after SAH**. No significant differences were observed between groups. (**B**) Time course of dynamic MAP changes until 180 minutes after SAH. MAP was significantly higher before and after SAH when animals were anesthetized with MMF as compared to chloral hydrate or isoflurane. SAH induced a Cushing response (transient increase of MAP due to increased ICP) in animals anesthetized with MMF while no such response was observed in the isoflurane or chloral hydrate groups. ICP and MAP were recorded continuously, averaged over 3 minutes, and displayed as means ± SEM (n = 5 in each group; *p < 0.05 vs. isoflurane, #*p *< 0.05 vs. chloral hydrate; Kruskal-Wallis ANOVA on ranks followed by Dunn's post-hoc test).

There was no significant difference in ICP (values around 5 mmHg) between the three groups under physiological conditions. SAH resulted in an immediate increase in ICP to a peak of 42 ± 8, 28 ± 4 and 61 ± 17 mmHg in the chloral hydrate, isoflurane and MMF group, respectively (Figure 1A). This peak was followed by a plateau of 15-20 mmHg that was reached after 15 minutes in all groups.

Under physiological conditions animals subjected to MMF anesthesia exhibited a significantly (*p *< 0.05) higher MAP (~100 mmHg) compared to the groups anesthetized with chloral hydrate (~80 mmHg) or isoflurane (~70 mmHg). After SAH MAP remained significantly (*p *< 0.05) elevated in the MMF group (Figure 1B). Of note, the sudden rise in ICP after SAH triggered a Cushing reflex in MMF anesthetized animals: MAP acutely increased by ~20 mmHg (from 98 ± 7 to 118 ± 12 mmHg) and returned to baseline values 15 minutes later. In animals anesthetized with chloral hydrate or isoflurane, however, this expected physiological increase in blood pressure was not observed. On the contrary, MAP actually dropped by 20 mmHg and recovered only slowly thereafter (Figure 1B) indicating that chloral hydrate and isoflurane interfere with physiological blood pressure regulation.

Due to the higher MAP under physiological conditions, CPP was significantly (*p *< 0.05) higher in MMF anesthetized rats (~95 mmHg) compared to those animals which received chloral hydrate (~75 mmHg) or isoflurane (~65 mmHg). Following SAH, CPP decreased by ~30-45 mmHg in all experimental groups. In the MMF group, CPP returned to near baseline values of 88 ± 7 mmHg within 5 minutes after SAH. For chloral hydrate and isoflurane a constantly reduced CPP was observed after SAH (51 ± 7 and 41 ± 3 mmHg, respectively; *p *< 0.05 vs. MMF).

Anesthesia with chloral hydrate or MMF did not increase rCBF during the 3 hour observation time (7 ± 13% and 5 ± 6% rCBF increase vs. 30 min after initiation of anesthesia, respectively), while in isoflurane anesthetized rats rCBF increased by over 1/3 (35 ± 22% rCBF increase vs. 30 min after initiation of anesthesia).

**Figure 2:**
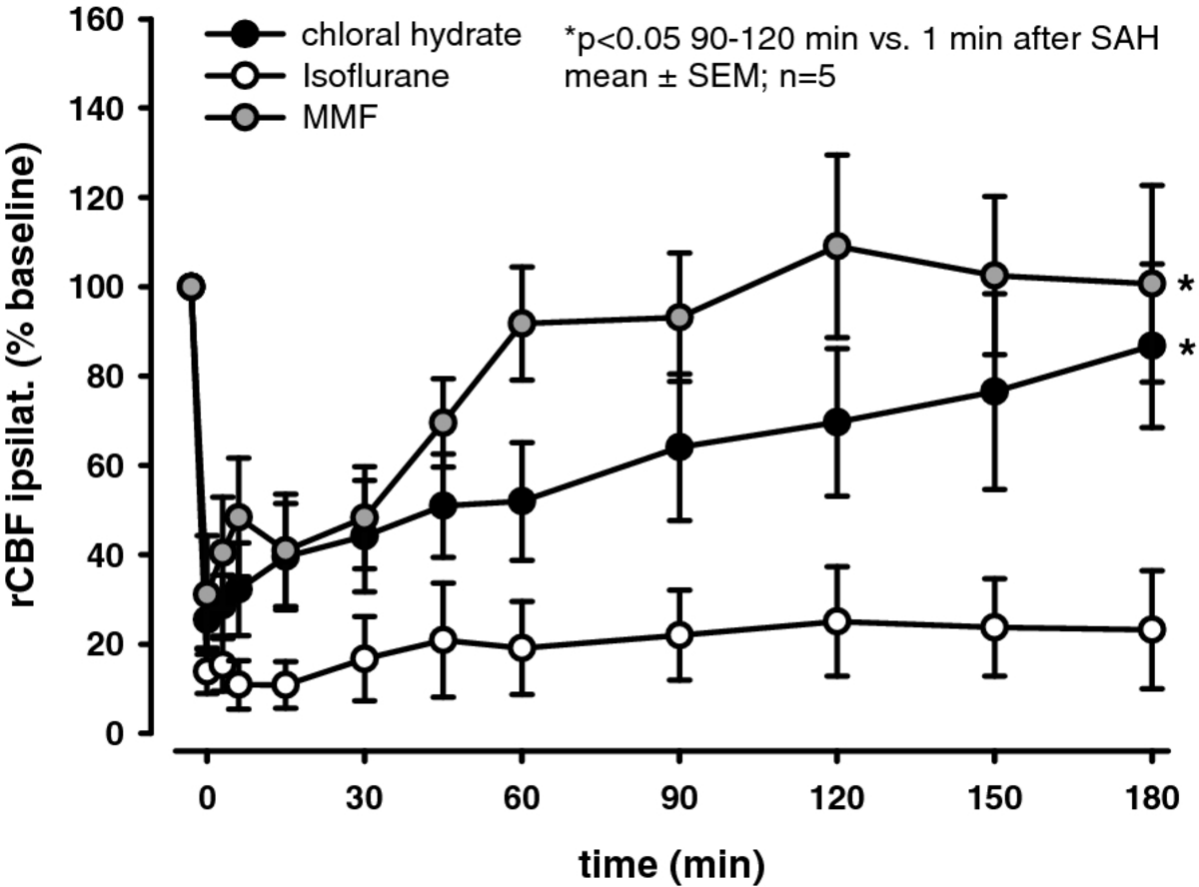
**Regional cerebral blood flow of the hemisphere ipsilateral to hemorrhage measured by laser Doppler fluxmetry until 180 minutes after SAH**. SAH resulted in an immediate reduction of rCBF in all groups with a significantly better recovery in the MMF group (*p < 0.05 vs. isoflurane). rCBF was recorded continuously, averaged over 3 minutes, and displayed as percentage (%) of pre-hemorrhagic baseline (means ± SEM; n = 5 in each group; Kruskal-Wallis ANOVA on ranks followed by Dunn's post-hoc test).

In all animals SAH resulted in a sharp decline of ipsilateral rCBF (*p *< 0.05) to ~10-30% of baseline (Figure 2). No recovery was observed in the isoflurane group while in MMF and chloral hydrate anesthetized rats rCBF recovered to almost baseline values (92 ± 13% and 87 ± 18%, respectively). Contralateral rCBF recovered within the 3 hours observation period in all groups.

### Brain water content (BWC)

**Figure 3:**
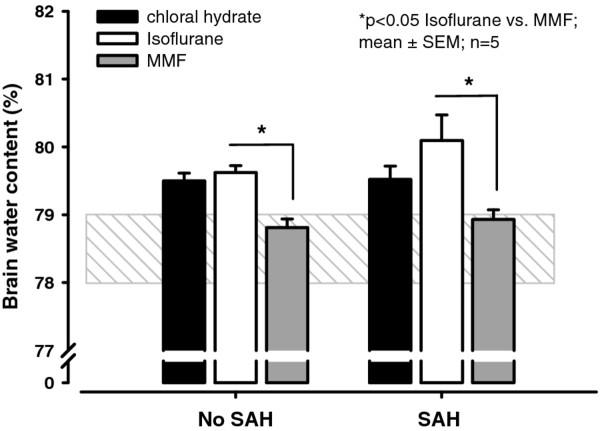
**Brain water content (BWC) 3 hours following SAH or in respective control animals**. Streaked gray area indicates the range of normal brain water content in animals not subjected to long-term anesthesia. BWC is within the normal range under MMF anesthesia while it is elevated in rats anesthetized with isoflurane (*p *< 0.05) or chloral hydrate (Control). Following SAH BWC increased only in animals anesthetized with isoflurane while it remained unchanged in animals receiving chloral hydrate or MMF. Values are presented as mean ± SEM for n = 5 in each group, *p < 0.05 vs. isoflurane (Kruskal-Wallis ANOVA on ranks followed by Dunn's Method).

Following 4.5 hours of anesthesia, brain water content was in the physiological range in the MMF group (78.8 ± 0.1%) but increased in animals anesthetized with chloral hydrate or isoflurane (*p *< 0.05; 79.5 ± 0.1% and 79.6 ± 0.1%, respectively; Figure 3) suggesting either opening of the blood brain barrier or, more likely, increasing cerebral blood volume due to anesthesia-induced vasodilation.

Three hours after induction of SAH brain water content was 79.5 ± 0.2%, 80.1 ± 0.4%, and 78.9 ± 0.1% in the chloral hydrate, isoflurane and MMF groups, respectively. SAH did not result in an increase in brain water content in chloral hydrate and MMF anesthetized animals (0% and 0.1% ΔBWC^pre/post^), while slight brain edema formation (0.5% ΔBWC^pre/post^) was observed in the isoflurane group (*p *< 0.05 vs. MMF; Figure 3).

### Mortality and re-bleeding after SAH

There was no mortality during surgical preparation and sham operated animals did not show any mortality.

**Figure 4:**
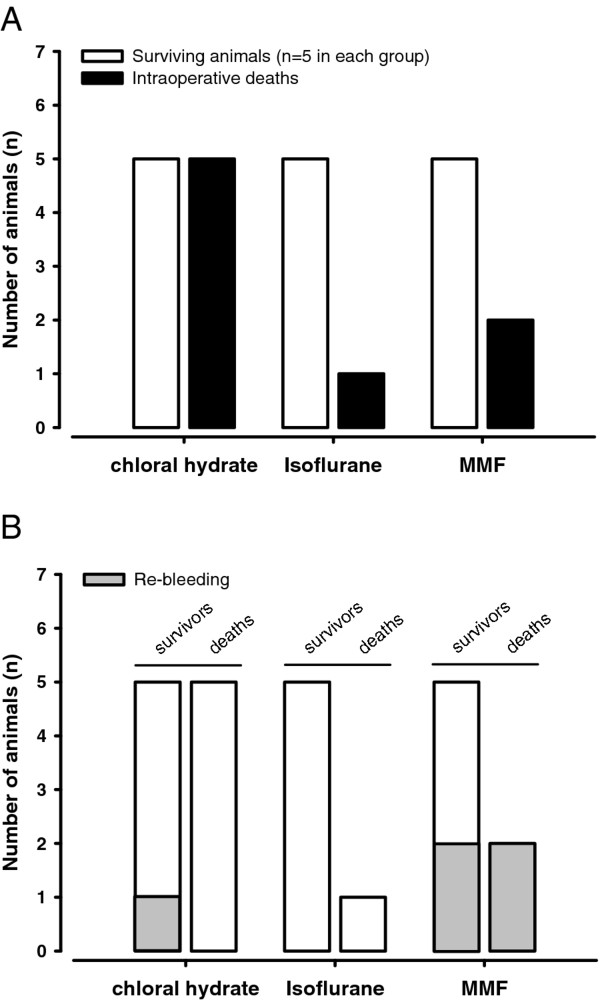
**(A) Total number of operated rats per group stratified by survival**. The number of animals per group was increased until n = 5 rats survived the 3 hour post-operative observation period. Mortality in animals anesthetized with chloral hydrate, isoflurane, or MMF was 50%, 17%, and 29%, respectively. **(B) **Number of animals which suffered a re-bleeding following SAH stratified by animals surviving (survivors) or not surviving (deaths) the post-operative observation period of 3 hours. Numbers of animals which died from anesthesia-induced complications are given in gray.

After SAH, animals anesthetized with chloral hydrate had the highest rate of intraoperative mortality (50%) followed by animals anesthetized with MMF (29%) and isoflurane (17%, Figure 4A).

Significant re-bleedings, which were identified by a recurrent ICP increase of > 20 mmHg, were detected in chloral hydrate (1/10, 10%) and MMF (4/7, 57%) anesthetized animals, in the latter contributing to the mortality in this group (Figure 4B). In total 8 out of the 38 animals used for the current study died prematurely resulting in n = 30 animals being included in the final analysis.

## Discussion

In the present study we evaluated the effect of three widely used and well standardized anesthesia protocols on pathophysiology and outcome following subarachnoid hemorrhage (SAH) in rats. Surprisingly, different anesthesia protocols resulted in completely different pathophysiologies following SAH. Isoflurane and chloral hydrate anesthesia resulted in low blood pressure, lack of the expected physiological post-ictal Cushing response, impaired recovery of post-hemorrhagic ischemia and no re-bleedings, while a combination of midazolam, medetomidine and fentanyl (MMF) maintained physiological blood pressure, allowed the rising ICP to trigger the expected increase in systemic blood pressure (Cushing reflex), resulted in complete recovery of post-hemorrhagic cerebral blood flow and caused a significant number of re-bleedings, an important characteristic of SAH in humans.

### Ventilation and arterial blood gases

Most agents used for small animal anesthesia cause a dose dependent depression of central respiratory activity leading to deterioration in arterial blood gases [20,21]. Therefore, particularly with longer duration experiments, intubation and mechanical ventilation is used to keep blood gases within physiological limits [5]. Animals in the isoflurane group experienced a gradual decrease in arterial pO_2 _(blood oxygenation) and, similarly to chloral hydrate, an increase in arterial pCO_2 _that required continuous adjustments of ventilation parameters. Airway irritation and impaired mucocilliary clearance have previously been described for inhalative agents like isoflurane and halothane [22,23] and most probably account for the reduced gas exchange and deterioration in blood gases. Keeping arterial paCO_2 _within physiological limits is particularly critical following pathological conditions, as carbon dioxide is not only a potent regulator of cerebrovascular tone [24] but hypercapnia and subsequent acidosis are known to increase ischemic brain injury [25,26]. Also, hypoxemia, a symptom of respiratory distress which is frequently observed after SAH [27,28], has been shown to increase the likelihood of a poor outcome [29-31]. In contrast to isoflurane and chloral hydrate the MMF regimen did not affect ventilation; pO_2 _and pCO_2 _remained in the physiological range without necessitating constant adjustment of ventilator settings. Therefore, in our hands, MMF anesthesia provided the most stable physiological conditions (especially in combination with mechanical ventilation), allowing prolonged, hassle-free surgery.

### MAP, ICP, CPP, and rCBF

Anesthesia with chloral hydrate or isoflurane caused a reduction of MAP, most likely due to their well known negative effect on cardiac function and vascular tone [20,32]. In contrast, MAP remained closest to physiological values of conscious rats (100-110 mmHg) [33] in the MMF group. The difference in MAP amongst the groups correlated with the ICP increase during SAH induction, which implies that the amount and severity of hemorrhage in this model is directly influenced by pre-hemorrhagic MAP. MAP monitoring is, therefore, an absolute prerequisite for the proper and reproducible induction of experimental SAH.

The acute stage of SAH is frequently accompanied by derangements in arterial blood pressure [3,31,34]. A hypertensive reaction, as exhibited in MMF animals, is described as central activation of vasomotor centers to restore cerebral perfusion in the presence of raised ICP, i.e. the well known Cushing reflex [35,36]. The lack of such a robust physiological reflex in the chloral hydrate and isoflurane group suggests that these agents severely affect the cardiovascular system, thereby altering post-hemorrhagic pathophysiology in an uncontrolled manner. Our data, therefore, indicate that studies on the pathophysiology of SAH under chloral hydrate or isoflurane anesthesia may need to be re-interpreted carefully, since there is a high probability that they may have been performed under a disturbed cardiovascular baseline.

Even more divergent effects of the three anesthesia protocols were observed when post-hemorrhagic rCBF was measured. Whilst rats anesthetized with MMF showed an almost complete recovery from SAH-induced ischemia due to high MAP and CCP, rCBF did not recover at all in animals receiving isoflurane anesthesia. Since isoflurane and chloral hydrate have a very similar suppressive effect on systemic blood pressure these findings suggest that isoflurane may have an additional vasodilatatory effect on cerebral vessels, as also suggested by others [37]. The mechanisms responsible for this effect seem to be a selective upregulation of nitric oxide synthesis in the brain by volatile anesthetics [38]. These findings suggest that the use of isoflurane and other volatile anesthetics should be discouraged when studying SAH.

### Brain water content

Our findings demonstrating increased brain water content in rats anesthetized with isoflurane are supported by previous studies where volatile anesthetics increased brain water content in healthy rats and dogs [11,39]. These findings are in agreement with the local vasodilatatory effects of isoflurane on the cerebral vasculature; vasodilation increases cerebral blood volume and hence brain water content [40-42].

Isoflurane anesthesia increases brain water content following brain injury, e.g. after experimental traumatic brain injury [11] and after focal cryogenic lesion [43] as also observed in the current study. This is most likely caused by the effect of isoflurane on cerebral blood volume (see above), however, brain damage due to prolonged post-hemorrhagic ischemia/hypoperfusion [44] would also be expected to be involved in the development of brain edema in animals anesthetized with isoflurane.

### Mortality

The two major determinants of post-hemorrhagic mortality in our study were lack of recovery of cerebral hypoperfusion and re-bleeding. Interestingly, the occurrence of hypoperfusion and re-bleeding depended exclusively on the anesthetic protocol used. Hypoperfusion-/ischemic-induced death only occurred after isoflurane and chloral hydrate anesthesia. While isoflurane seems to have a direct CBF-reducing effect (see above), chloral hydrate-induced death was, in most cases, due to episodes of arterial hypotension followed by cessation of rCBF following intraperitoneal re-application of chloral hydrate, as also reported in experimental stroke [45]. This specific problem may, however, be overcome by continuous application of chloral hydrate using a intraperitoneal catheter [46].

Mortality occurring in animals anesthetized with MMF was, in all cases, related to post-hemorrhagic re-bleedings, which are an integral part of the pathophysiology of SAH in humans. The underlying mechanisms of MMF-induced re-bleedings are the maintained MAP and CPP. High MAP and CPP, although beneficial for cerebral perfusion, increase the risk for re-hemorrhages [47] with a high mortality rate [48], as also suggested by our findings. Hence, using MMF for anesthesia during experimental SAH in rats reproduces an important component of SAH in patient, i.e. delayed re-bleedings, which is usually missing when using when chloral hydrate or isoflurane. Accordingly, only when rats were anesthetized with MMF the pathophysiology of SAH was properly reproduced by the endovascular perforation model.

### Handling for general anesthesia in rats

All three protocols proved to be suitable for surgical anesthesia, however, only MMF appears to not have any negative effect on the pulmonary system, thereby resulting in a far more stable maintenance of arterial pCO_2 _and pO_2 _with fewer interventions by the experimentalist compared to isoflurane and chloral hydrate. When considering that MMF anesthesia can be terminated by injection of respective antagonists and that anesthesia under chloral hydrate is difficult to judge and to terminate [49], it seems fair to conclude that MMF is superior to the other two investigated protocols.

## Conclusion

We investigated if and how three widely used and well standardized anesthesia protocols [isoflurane, chloral hydrate, and a combination of midazolam, medetomidine, and fentanyl (MMF)] affect pathophysiology and outcome following experimental SAH in rats. Our experiments demonstrate that when rats are anesthetized with MMF key properties (re-bleedings, Cushing reflex, moderate mortality) known to be integral parts of the pathophysiology of SAH in humans [47,48,50] are replicated by the endovascular perforation model of experimental SAH. Our findings demonstrate that the choice of anesthesia can have a significant impact on animal models of disease and suggest that MMF should be used for future studies using the endovascular perforation model of SAH.

## Competing interests

The authors declare that they have no competing interests.

## Authors' contributions

KH carried out the SAH experiments, performed data analysis, and drafted the manuscript. RT participated in the design and coordination of the study and supervised the experiments. KS participated in the design of the study and edited the manuscript. ET supported KH in performing the experiments and analyzing the data. NP initiated, designed, and coordinated the study and finalized the manuscript. All authors read and approved the final manuscript.

## References

[B1] Bederson JB Germano IM Guarino L Cortical blood flow and cerebral perfusion pressure in a new noncraniotomy model of subarachnoid hemorrhage in the rat Stroke 1995 26 1086 1091 10.1161/01.STR.26.6.1086 7762027

[B2] Prunell GF Mathiesen T Diemer NH Svendgaard NA Experimental subarachnoid hemorrhage: subarachnoid blood volume, mortality rate, neuronal death, cerebral blood flow, and perfusion pressure in three different rat models Neurosurgery 2003 52 165 175 12493115 10.1097/00006123-200301000-00022

[B3] Schwartz AY Masago A Sehba FA Bederson JB Experimental models of subarachnoid hemorrhage in the rat: a refinement of the endovascular filament model J Neurosci Methods 2000 96 161 167 10.1016/S0165-0270(00)00156-4 10720681

[B4] Zausinger S Thal SC Kreimeier U Messmer K Schmid-Elsaesser R Hypertonic fluid resuscitation from subarachnoid hemorrhage in rats Neurosurgery 2004 55 679 686 10.1227/01.NEU.0000134558.28977.EE 15335436

[B5] Zausinger S Baethmann A Schmid-Elsaesser R Anesthetic methods in rats determine outcome after experimental focal cerebral ischemia: mechanical ventilation is required to obtain controlled experimental conditions Brain Res Brain Res Protoc 2002 9 112 121 10.1016/S1385-299X(02)00138-1 12034330

[B6] Bederson JB Levy AL Ding WH Kahn R DiPerna CA Jenkins AL III Vallabhajosyula P: Acute vasoconstriction after subarachnoid hemorrhage Neurosurgery 1998 42 352 360 10.1097/00006123-199802000-00091 9482187

[B7] Jackowski A Crockard A Burnstock G Russell RR Kristek F The time course of intracranial pathophysiological changes following experimental subarachnoid haemorrhage in the rat J Cereb Blood Flow Metab 1990 10 835 849 10.1038/jcbfm.1990.140 2211877

[B8] Piepgras A Thome C Schmiedek P Characterization of an anterior circulation rat subarachnoid hemorrhage model Stroke 1995 26 2347 2352 10.1161/01.STR.26.12.2347 7491662

[B9] Prunell GF Mathiesen T Svendgaard NA Experimental subarachnoid hemorrhage: cerebral blood flow and brain metabolism during the acute phase in three different models in the rat Neurosurgery 2004 54 426 436 10.1227/01.NEU.0000103670.09687.7A 14744290

[B10] Statler KD Alexander H Vagni V Dixon CE Clark RS Jenkins L Kochanek PM Comparison of seven anesthetic agents on outcome after experimental traumatic brain injury in adult, male rats J Neurotrauma 2006 23 97 108 10.1089/neu.2006.23.97 16430376

[B11] Stover JF Sakowitz OW Kroppenstedt SN Thomale UW Kempski OS Flugge G Unterberg AW Differential effects of prolonged isoflurane anesthesia on plasma, extracellular, and CSF glutamate, neuronal activity, 125I-Mk801 NMDA receptor binding, and brain edema in traumatic brain-injured rats Acta Neurochir (Wien) 2004 146 819 830 15254804 10.1007/s00701-004-0281-9

[B12] Scholler K Trinkl A Klopotowski M Thal SC Plesnila N Trabold R Hamann GF Schmid-Elsaesser R Zausinger S Characterization of microvascular basal lamina damage and blood-brain barrier dysfunction following subarachnoid hemorrhage in rats Brain Res 2007 1142 237 246 17303089 10.1016/j.brainres.2007.01.034

[B13] Torok E Klopotowski M Trabold R Thal SC Plesnila N Scholler K Mild hypothermia (33 degrees C) reduces intracranial hypertension and improves functional outcome after subarachnoid hemorrhage in rats Neurosurgery 2009 65 352 359 10.1227/01.NEU.0000345632.09882.FF 19625915

[B14] Barry KJ Gogjian MA Stein BM Small animal model for investigation of subarachnoid hemorrhage and cerebral vasospasm Stroke 1979 10 538 541 10.1161/01.STR.10.5.538 505495

[B15] Doczi T Joo F Adam G Bozoky B Szerdahelyi P Blood-brain barrier damage during the acute stage of subarachnoid hemorrhage, as exemplified by a new animal model Neurosurgery 1986 18 733 739 10.1227/00006123-198606000-00010 3736802

[B16] Germano A d'Avella D Imperatore C Caruso G Tomasello F Time-course of blood-brain barrier permeability changes after experimental subarachnoid haemorrhage Acta Neurochir (Wien) 2000 142 575 580 10.1007/s007010050472 10898366

[B17] Miyagi Y Carpenter RC Meguro T Parent AD Zhang JH Upregulation of rho A and rho kinase messenger RNAs in the basilar artery of a rat model of subarachnoid hemorrhage J Neurosurg 2000 93 471 476 10.3171/jns.2000.93.3.0471 10969946

[B18] Scholler K Feiler S Anetsberger S Kim SW Plesnila N Contribution of Bradykinin Receptors to the Development of Secondary Brain Damage After Experimental Subarachnoid Hemorrhage Neurosurgery 2011 in press 10.1227/NEU.0b013e31820a0024 21242838

[B19] Feiler S Friedrich B Scholler K Thal SC Plesnila N Standardized induction of subarachnoid hemorrhage in mice by intracranial pressure monitoring J Neurosci Methods 2010 190 164 170 10.1016/j.jneumeth.2010.05.005 20457182

[B20] Field KJ White WJ Lang CM Anaesthetic effects of chloral hydrate, pentobarbitone and urethane in adult male rats Lab Anim 1993 27 258 269 10.1258/002367793780745471 8366672

[B21] Wixson SK White WJ Hughes HC Jr Lang CM Marshall WK The effects of pentobarbital, fentanyl-droperidol, ketamine-xylazine and ketamine-diazepam on arterial blood pH, blood gases, mean arterial blood pressure and heart rate in adult male rats Lab Anim Sci 1987 37 736 742 3125387

[B22] Cervin A Lindberg S Changes in mucociliary activity may be used to investigate the airway-irritating potency of volatile anaesthetics Br J Anaesth 1998 80 475 480 9640154 10.1093/bja/80.4.475

[B23] Doi M Ikeda K Airway irritation produced by volatile anaesthetics during brief inhalation: comparison of halothane, enflurane, isoflurane and sevoflurane Can J Anaesth 1993 40 122 126 10.1007/BF03011308 8443850

[B24] Madden JA The effect of carbon dioxide on cerebral arteries Pharmacol Ther 1993 59 229 250 10.1016/0163-7258(93)90045-F 8278463

[B25] Browning JL Heizer ML Widmayer MA Baskin DS Effects of halothane, alpha-chloralose, and pCO2 on injury volume and CSF beta-endorphin levels in focal cerebral ischemia Mol Chem Neuropathol 1997 31 29 42 10.1007/BF02815158 9271003

[B26] Katsura K Kristian T Smith ML Siesjo BK Acidosis induced by hypercapnia exaggerates ischemic brain damage J Cereb Blood Flow Metab 1994 14 243 250 10.1038/jcbfm.1994.31 8113321

[B27] Fontes RB Aguiar PH Zanetti MV Andrade F Mandel M Teixeira MJ Acute neurogenic pulmonary edema: case reports and literature review J Neurosurg Anesthesiol 2003 15 144 150 10.1097/00008506-200304000-00013 12658001

[B28] Vespa PM Bleck TP Neurogenic pulmonary edema and other mechanisms of impaired oxygenation after aneurysmal subarachnoid hemorrhage Neurocrit Care 2004 1 157 170 10.1385/NCC:1:2:157 16174911

[B29] Claassen J Vu A Kreiter KT Kowalski RG Du EY Ostapkovich N Fitzsimmons BF Connolly ES Mayer SA Effect of acute physiologic derangements on outcome after subarachnoid hemorrhage Crit Care Med 2004 32 832 838 10.1097/01.CCM.0000114830.48833.8A 15090970

[B30] Kahn JM Caldwell EC Deem S Newell DW Heckbert SR Rubenfeld GD Acute lung injury in patients with subarachnoid hemorrhage: incidence, risk factors, and outcome Crit Care Med 2006 34 196 202 10.1097/01.CCM.0000194540.44020.8E 16374174

[B31] Wartenberg KE Schmidt JM Claassen J Temes RE Frontera JA Ostapkovich N Parra A Connolly ES Mayer SA Impact of medical complications on outcome after subarachnoid hemorrhage Crit Care Med 2006 34 617 623 16521258 10.1097/01.ccm.0000201903.46435.35

[B32] Imai A Steffey EP Farver TB Ilkiw JE Assessment of isoflurane-induced anesthesia in ferrets and rats Am J Vet Res 1999 60 1577 1583 10622172

[B33] Head GA McCarty R Vagal and sympathetic components of the heart rate range and gain of the baroreceptor-heart rate reflex in conscious rats J Auton Nerv Syst 1987 21 203 213 10.1016/0165-1838(87)90023-3 3450695

[B34] Marshman LA Cushing's'variant' response (acute hypotension) after subarachnoid hemorrhage. Association with moderate intracranial tensions and subacute cardiovascular collapse Stroke 1997 28 1445 1450 10.1161/01.STR.28.7.1445 9227698

[B35] Dickinson CJ Reappraisal of the Cushing reflex: the most powerful neural blood pressure stabilizing system Clin Sci (Lond) 1990 79 543 550 2176941 10.1042/cs0790543

[B36] Cushing H Concerning a definite regulatory mechanism of the vasomotor centre which controls blood pressure during cerebral compression Bull Johns Hopkins Hosp 1901 12 290 292

[B37] Matta BF Heath KJ Tipping K Summors AC Direct cerebral vasodilatory effects of sevoflurane and isoflurane Anesthesiology 1999 91 677 680 10.1097/00000542-199909000-00019 10485778

[B38] Baumane L Dzintare M Zvejniece L Meirena D Lauberte L Sile V Kalvinsh I Sjakste N Increased synthesis of nitric oxide in rat brain cortex due to halogenated volatile anesthetics confirmed by EPR spectroscopy Acta Anaesthesiol Scand 2002 46 378 383 10.1034/j.1399-6576.2002.460408.x 11952436

[B39] Schettini A Furniss WW Brain water and electrolyte distribution during the inhalation of halothane Br J Anaesth 1979 51 1117 1124 10.1093/bja/51.12.1117 526377

[B40] Flynn NM Buljubasic N Bosnjak ZJ Kampine JP Isoflurane produces endothelium-independent relaxation in canine middle cerebral arteries Anesthesiology 1992 76 461 467 10.1097/00000542-199203000-00021 1539859

[B41] Iida H Ohata H Iida M Watanabe Y Dohi S Isoflurane and sevoflurane induce vasodilation of cerebral vessels via ATP-sensitive K + channel activation Anesthesiology 1998 89 954 960 10.1097/00000542-199810000-00020 9778013

[B42] McPherson RW Kirsch JR Tobin JR Ghaly RF Traystman RJ Cerebral blood flow in primates is increased by isoflurane over time and is decreased by nitric oxide synthase inhibition Anesthesiology 1994 80 1320 1327 10.1097/00000542-199406000-00020 7516627

[B43] Smith AL Marque JJ Anesthetics and cerebral edema Anesthesiology 1976 45 64 72 10.1097/00000542-197607000-00012 937753

[B44] Murr R Berger S Schurer L Kempski O Staub F Baethmann A Relationship of cerebral blood flow disturbances with brain oedema formation Acta Neurochir Suppl (Wien) 1993 59 11 17 7508676 10.1007/978-3-7091-9302-0_2

[B45] Theodorsson A Holm L Theodorsson E Modern anesthesia and peroperative monitoring methods reduce per- and postoperative mortality during transient occlusion of the middle cerebral artery in rats Brain Res Brain Res Protoc 2005 14 181 190 10.1016/j.brainresprot.2005.01.002 15795172

[B46] Henninger N Heimann A Kempski O Electrophysiology and neuronal integrity following systemic arterial hypotension in a rat model of unilateral carotid artery occlusion Brain Res 2007 1163 119 129 17632088 10.1016/j.brainres.2007.06.006

[B47] Fujii Y Takeuchi S Sasaki O Minakawa T Koike T Tanaka R Ultra-early rebleeding in spontaneous subarachnoid hemorrhage J Neurosurg 1996 84 35 42 10.3171/jns.1996.84.1.0035 8613833

[B48] Broderick JP Brott TG Duldner JE Tomsick T Leach A Initial and recurrent bleeding are the major causes of death following subarachnoid hemorrhage Stroke 1994 25 1342 1347 10.1161/01.STR.25.7.1342 8023347

[B49] Silverman J Muir WW III A review of laboratory animal anesthesia with chloral hydrate and chloralose Lab Anim Sci 1993 43 210 216 8355479

[B50] Grote E Hassler W The critical first minutes after subarachnoid hemorrhage Neurosurgery 1988 22 654 661 10.1227/00006123-198804000-00006 3287211

